# Association of remnant cholesterol with CVD incidence: a general population cohort study in Southwest China

**DOI:** 10.3389/fcvm.2023.1286286

**Published:** 2023-11-27

**Authors:** Chengxi Liu, Mi Dai, Kunming Tian, Shiyu Zhou, Lei Luo, Zhiying Zeng, Xuelian Yan, Ying Xiao, Yiying Wang, Renli Deng, Xiuhong Lei, Tao Liu

**Affiliations:** ^1^ Department of Anesthesiology, The Second Affiliated Hospital, Hengyang Medical School, University of South China, Hengyang, China; ^2^ Department of Preventive Medicine, School of Public Health, Zunyi Medical University, Zunyi, China; ^3^ The Third Affiliated Hospital of Zunyi Medical University (The First People's Hospital of Zunyi), Zunyi, China; ^4^ Department of Nursing, Affiliated Hospital of Zunyi Medical University, Zunyi, China; ^5^ Department of Chronic Disease Prevention and Control, Guizhou Disease Prevention and Control, Guiyang, China

**Keywords:** remnant cholesterol, cardiovascular disease, general population, cohort study, China

## Abstract

**Background:**

Emerging evidence has indicated that remnant cholesterol (RC) could predict cardiovascular disease (CVD) incidence. Nevertheless, the relationship between RC and CVD risk, especially within the general Chinese population, remains scarce.

**Objective:**

The present research aimed to assess whether RC concentrations and CVD outcomes in general Chinese adults are related.

**Methods:**

The Cox proportional hazard model was established to explore the relationship between RC and the outcomes of CVD and CVD subgroups. A restricted cubic spline (RCS) was utilized to investigate the dose–response connection between RC and the risk of CVD outcomes, and the ROC curve was used to calculate the corresponding cutoff values. Moreover, stratified analysis was conducted to investigate the potential effect modification in the association between RC and CVD outcomes.

**Results:**

Significant positive associations were found between elevated categorical RC and increased risk of CVD (HR Q4, 1.80; 95% CI 1.15–2.79; *P*-value = 0.008), atherosclerotic cardiovascular disease (HR Q4, 2.00; 95% CI 1.22–3.27; *P*-value = 0.007), stroke (HR Q4, 1.66; 95% CI 1.02–2.69; *P*-value = 0.040), and ischemic stroke (HR Q4, 1.87, 95% CI 1.08–3.25; *P*-value = 0.034), respectively. Our study suggested that the incidence of CVD outcomes increased when RC levels were above 0.75 mmol/L. Importantly, the CVD risks related to RC were more likely to be those found in subjects aged above 60 years, women, subjects with BMI <24 kg/m^2^, and subjects with hypertension and unhealthy diet patterns.

**Conclusions:**

Aberrant high level of RC is associated with elevated CVD risk, independent of low-density lipoprotein cholesterol (LDL-C). Our data reveal urgent primary prevention for subjects with high RC levels to a low incidence of CVD, especially for the elderly, women, and those with hypertension and unhealthy diet patterns.

## 1. Introduction

Cardiovascular disease (CVD) is the dominant cause of global mortality (1), accounting for 40% of deaths in China (2). More importantly, developing countries, such as China and India, suffer the enormous burden of CVD (3). Therefore, early identification and management of patients with high CVD risk are critical for planning effective interventions for the susceptible population. It is well known that dyslipidemia greatly influences the pathogenesis of atherosclerotic cardiovascular disease (ASCVD). The association between low-density lipoprotein cholesterol (LDL-C) and ASCVD has been extensively studied (4, 5). Lowering plasma LDL-C levels has been recommended as a priority measure to prevent ASCVD for decades (6, 7). Unfortunately, although statins or other lipid-lowering drugs have controlled the LDL-C levels to the target range, a significant residual risk of CVD remains (8, 9). Therefore, the residual plasma lipid may partially contribute to the residual CVD risk. Emerging evidence has indicated that the deposition of RC on arterial walls may induce atherosclerosis.

Remnant cholesterol (RC), a critical member of triglyceride-rich lipoproteins (TRLs), which are partially lipidated by lipoprotein lipase, generally consists of very low-density lipoproteins (VLDLs), intermediate-density lipoproteins (IDLs), and chylomicron residues (10). Mounting evidence indicates that RC, besides LDL cholesterol, is a primary causative agent of ASCVD (11, 12). Recent evidence suggests that RC was strongly related to cardiovascular events and total mortality (11–17). However, most studies investigating the connection between RC and CVD were carried out in Western populations, and the evidence for the Asian population is scarce (13, 18). Several studies have addressed the relationship between RC and CVD events in peritoneal dialysis (PD) (19), percutaneous coronary intervention (PCI) (20), type 2 diabetes, and incident diabetic nephropathy patients in China. Currently, quite limited studies have been established regarding the risk of RC with CVD among the general Chinese population. Only one longitudinal cohort in rural northeast China was explored to study the association between RC and CVD (14). Considering the high variation of demographic differences, more evidence is needed to identify the risk of RC for CVD using the representative population. In addition, an acceptable range or cutoff value of RC is also crucial for early prevention and treatment for the high-risk population. Also, these limitations have yet to be explored.

This investigation was carried out to elaborate on the relationship between RC and CVD in a large-sample prospective cohort study that contains a representative southwest China population. We systematically explored the relationship between RC and risks of CVD and its subtypes, and the cutoff value for the above associations was also identified. Stratified analysis was further performed to explore the modification factors impacting the RC and CVD relationships. Our study is of great scientific proof to identify the economic biomarker for diagnosing the population with high risk for CVD.

## 2. Methods

### 2.1. Study population

**Figure 1:**
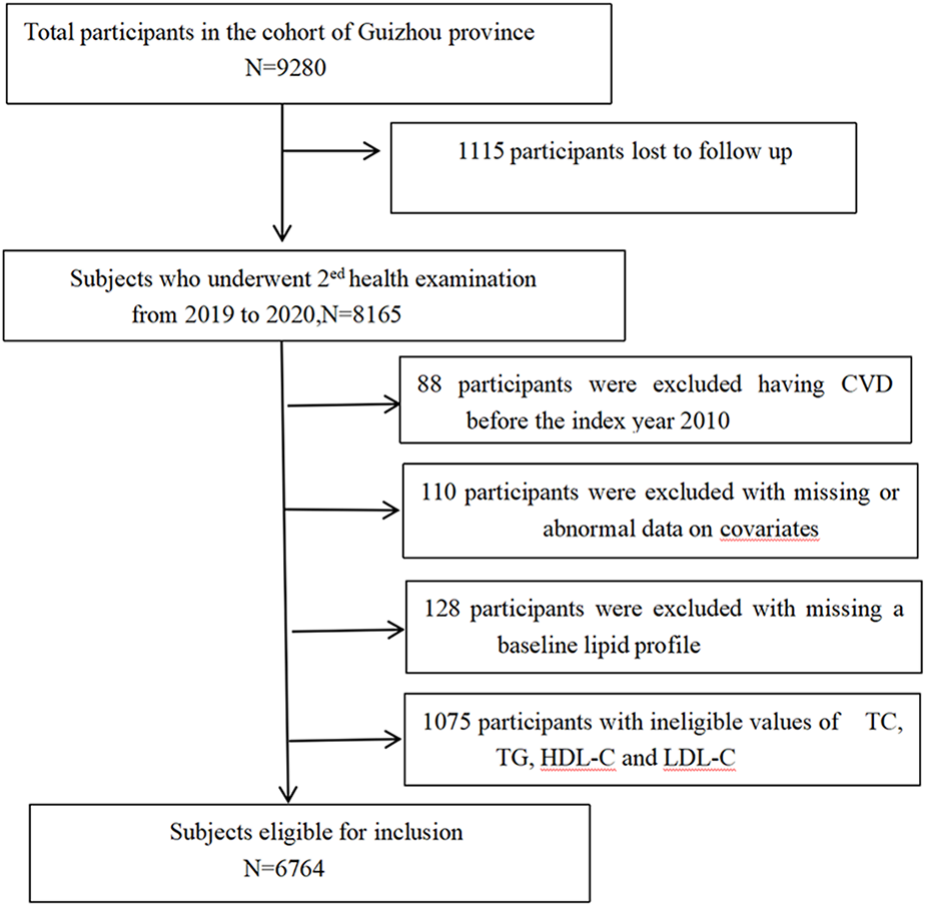
Flowchart of the study sample.

This is a multistage and randomly stratified cluster sample design including 9,280 adults (age ≥18 years) enlisted between October 2010 and August 2012 in Guizhou Province's 48 townships across 12 districts. A total of 8,165 study participants completed at least one follow-up visit in this 10-year follow-up study. The following individuals were excluded from this study: (1) those who were diagnosed with baseline CVD [*n* = 88, incidence of combined ischemic heart disease (IHD) and stroke]; (2) those with missing data of baseline lipid (*n* = 1,203); and (3) those with a lack of information on population baseline covariates (*n* = 110). Finally, 6,764 subjects were included in this study. Figure 1 depicts the study flow. To carry out this investigation, we received permission from the Guizhou Center for Disease Control and Prevention's institutional review committee (No. S2017-02). Each participant signed an informed consent form. The principles of the Declaration of Helsinki were followed in this investigation.

### 2.2. Laboratory methods

Participants providing venous blood samples were instructed to fast overnight for at least 8 h to measure triglyceride (TG), total cholesterol (TC), LDL-C, and HDL cholesterol (HDL-C) levels. A fully automated hematology analyzer (Sysmex, XN-9000, Japan) was used to detect blood parameters, and all tests were conducted by professional laboratory personnel. All testing equipment met quality control requirements. RC was obtained through the following formula: RC = TC-LDL-C‒HDL-C (21).

### 2.3. Outcome ascertainment

The main terminus was the composite of CVD events—including IHD and stroke. IHD was recognized as sudden cardiac death (death within 1 h of commencement, cardiac arrest, or sudden collapse without 1 h of symptoms), definite or probable myocardial infarction, and angina pectoris (22). A stroke was described as a focal nerve disease that developed suddenly and persisted for at least 24 h or till death. The specific incidence of IS and IHD was also documented. Participants were defined as ASCVD if they had a definite or probable myocardial infarction, coronary death, and stroke. The information to identify endpoints was derived from self-reported previous diagnoses and medical records or extracted from the Death Registration Information System. ICD-10, or the International Classification of Diseases, Tenth Revision, was used to categorize and encode the diagnosis. IHD is I20.x–125.x, stroke is I60.x–I69.x, and IS is I61.x–I68.x. ASCVD is ICD-9 410–414, 430–438, and 440.

### 2.4. Other covariates

Covariate information, including age, sex, BMI, LDL-C, excessive drinking (yes or no), current smoking status (yes or no), physical activity (never, 1–2 days/week, ≥3 days/week), insufficient cereal intake (yes or no), insufficient vegetable and fruit intake (yes or no), excessive meat intake (yes or no), bean product intake (yes or no), animal entrail intake (yes or no), lipid-lowering drug use (yes or no), diabetes mellitus (yes or no), and hypertension (yes or no), was referenced from previous literature studies and derived from questionnaires used by the interviewer face to face at baseline. According to the 2016 Dietary Guidelines for Chinese Residents (23), insufficient cereal intake was defined as daily intake of cereals <250 g, insufficient vegetable and fruit intake was defined as daily vegetable intake <300 g and daily fruit intake <200 g, excessive red meat intake was defined as daily meat intake >75 g, and excessive alcohol consumption was defined as >25 g/day for men and >15 g/day for women. Anthropometric measurements were used by skilled professionals to obtain data on height, weight, and blood pressure (BP). BMI was calculated by dividing weight (in kilograms) by the square of height (in meters). Based on the recommendation of the Working Group on Obesity in China (24), low weight, normal weight, overweight, and obesity were defined as BMI <18.5 kg/m^2^, BMI 18.5–23.9 kg/m^2^, BMI 24.0–27.9 kg/m^2^, and BMI ≥28 kg/m^2^, respectively. BP was calculated as the average of three readings using the same brand of an electronic sphygmomanometer. Participants were defined as having hypertension if they met the following criteria of JNC 7 (25): (1) self-reported diagnosis of hypertension or intake of hypotensive drugs; and/or (2) systolic blood pressure of 140 mmHg or higher and diastolic blood pressure of 90 mmHg or higher. Diabetes was defined by the American Diabetes Association diagnostic criterion (26): (1) self-reported diagnosis of diabetes, (2) FPG of 7.0 mmol/L (126 mg/dL) or higher, (3) 2-h glucose of 11.1 mmol/L (200 mg/dL) or higher, or (4) HbA1c concentration of higher than 6.5%.

### 2.5. Statistical analysis

Normally distributed variables and skewed data were presented as means ± standard deviations (SDs) and medians (25%, 75%), respectively. RC levels divided all subjects into four subgroups (Quartile 1, Quartile 2, Quartile 3, and Quartile 4). The difference among baseline characteristics of groups was compared through the Kruskal–Wallis test. Categorical data were presented as *n* (%) and compared utilizing the *χ*^2^ test.

The follow-up time was calculated and expressed as the interval between enrollment in the cohort and the incident CVD outcomes or the last visit time, whichever came first. Cox proportional hazard models were fitted to evaluate hazards ratios (HRs) and 95% confidence intervals (CIs) for the associations of RC (considered as both categorical and continuous variables) with CVD, ASCVD, CHD, stroke, and IS events. Schoenfeld residuals were utilized to investigate the proportionality of hazards per variable. Potential confounding factors were selected according to previous studies regarding the relation of RC with CVD events and biological plausibility. Three adjustment models were fitted for Cox analysis: (1) Model 1—adjusted for age and sex; (2) Model 2—Model 1 plus present smoking status, excessive drinking status, physical activity, insufficient cereal intake, insufficient vegetable and fruit intake, excessive meat intake, bean product intake, and animal entrail intake; (3) Model 3—Model 2 plus BMI, history of lipid-lowering drug use, LDL-C, diabetes, and hypertension. The *p*-value was obtained by assigning the median value as a continuous variable to each category. The dose–response relationship of RC with the risk of CVD outcomes was investigated using RCS, and the ROC curve was constructed to calculate the cutoff value to provide evidence for early screening of the susceptible population. Stratified analyses were further carried out to explore the potential effect modification by baseline age (≤60 years vs. >60 years), sex, current smoking status (yes or no), BMI subgroups (<24 kg/m^2^ vs. ≥24 kg/m^2^), insufficient vegetable and fruit intake (yes or no), excessive meat intake (yes or no), bean product intake (yes or no), animal entrail intake (yes or no), and hypertension (yes or no) on the association between different RC levels (Quartile 1 as the reference) and risks of CVD outcomes.

All statistical analyses were performed using SPSS version 25.0 and R 4.1.1, and statistical significance was defined by setting the two-sided *P*-value at 0.05.

## 3. Results

### 3.1. Baseline characteristics

We finally included 6,764 subjects for the present study, and Table 1 presents the baseline characteristics. Based on the RC concentration, we divided all subjects into four groups: Quartile 1 (RC ≤0.25 mmol/L), Quartile 2 (RC 0.25–0.67 mmol/L), Quartile 3 (RC 0.67–1.00 mmol/L), and Quartile 4 (RC >1.00 mmol/L). Among all participants, 47.7% were men; the mean age and mean BMI were 44.81 *± *15.09 years and 22.88 ± 3.25 kg/m^2^, respectively. The prevalence rates of hypertension and diabetes were 27.0% and 8.9%, respectively. Subjects with higher levels of RC tended to be older than those with low levels and more likely to be present smokers, excessive drinkers, diabetes, and hypertension. Moreover, individuals who consumed animal entrails, excessive meat, fewer vegetables and fruits, and insufficient cereals have higher RC levels. Meanwhile, serum levels of TC and TG increased along with the level of RC, while sex did not differ significantly across different groups.

**Table 1:** Basic characteristics of the participants according to different groups of remnant cholesterol.

Character	Total (*n* = 6,764)	Quartile 1 (*n* = 1,708)	Quartile 2 (*n* = 1,667)	Quartile 3 (*n* = 2,065)	Quartile 4 (*n* = 1,324)	*P*
Age (years)	44.81 ± 15.09	42.81 ± 15.20	44.25 ± 15.52	46.15 ± 14.93	46.02 ± 14.29	<0.001
BMI (kg/m^2^)	22.88 ± 3.25	22.70 ± 3.14	22.71 ± 3.14	23.06 ± 3.40	22.88 ± 3.29	0.002
Sex, *n* (%)						0.441
Male	3,228 (47.7)	803 (47.0)	806 (48.4)	966 (46.8)	653 (49.3)	
Female	3,536 (52.3)	905 (53.0)	861 (51.6)	1,099 (53.2)	671 (50.7)	
Current smoking, *n* (%)						0.002
Yes	1,970 (29.1)	506 (29.6)	455 (27.3)	659 (31.9)	350 (26.4)	
No	4,794 (70.9)	1,202 (70.4)	1,212 (72.7)	1,406 (68.1)	974 (73.6)	
Excessive drinking, *n* (%)						0.015
Yes	722 (10.7)	183 (10.7)	158 (9.5)	255 (12.3)	126 (9.5)	
No	6,042 (89.3)	1,525 (89.3)	1,509 (90.5)	1,810 (87.7)	1,198 (90.5)	
Physical activity, *n* (%)						<0.001
Never	6,215 (91.9)	1,539 (90.1)	1,586 (95.1)	1,877 (90.9)	1,213 (91.6)	
1–2 days per week	139 (2.1)	53 (3.1)	21 (1.3)	52 (2.5)	13 (1.0)	
≥3 days per week	410 (6.0)	116 (6.8)	60 (3.6)	136 (6.6)	98 (7.4)	
Inadequate cereal intake, *n* (%)						0.013
Yes	1,108 (16.4)	292 (17.1)	255 (15.3)	311 (15.1)	250 (18.9)	
No	5,656 (83.6)	1,416 (82.9)	1,412 (84.7)	1,754 (84.9)	1,074 (81.1)	
Inadequate vegetable and fruit intake, *n* (%)						<0.001
Yes	3,327 (49.2)	829 (48.5)	813 (48.8)	963 (46.6)	722 (54.5)	
No	3,437 (50.8)	879 (51.5)	854 (51.2)	1,102 (53.4)	602 (45.5)	
Excessive meat intake, *n* (%)						<0.001
Yes	2,518 (37.2)	691 (40.5)	554 (33.2)	851 (41.2)	422 (31.9)	
No	4,246 (62.8)	1,017 (59.5)	1,113 (66.8)	1,214 (58.8)	902 (68.1)	
Bean products intake, *n* (%)						<0.001
Yes	4,751 (70.2)	1,329 (77.8)	1,019 (61.1)	1,565 (75.8)	838 (63.3)	
No	2,013 (29.8)	379 (22.2)	648 (38.9)	500 (24.2)	486 (36.7)	
Animal entrail intake, *n* (%)						<0.001
Yes	3,294 (48.7)	906 (53.0)	734 (44.0)	1,154 (55.9)	500 (37.8)	
No	3,470 (51.3)	802 (47.0)	933 (56.0)	911 (44.1)	824 (62.2)	
Lipid-lowering drug use, *n* (%)						0.057
Yes	58 (0.9)	12 (0.7)	8 (0.5)	20 (1.0)	18 (1.4)	
No	6,706 (99.1)	1,696 (99.3)	1,659 (99.5)	2,045 (99.0)	1,306 (98.6)	
Diabetes, *n* (%)						<0.001
Yes	604 (8.9)	118 (6.9)	132 (7.9)	228 (11.0)	126 (9.5)	
No	6,160 (91.1)	1,590 (93.1)	1,535 (92.1)	1,837 (89.0)	1,198 (90.5)	
Hypertension, *n* (%)						<0.001
Yes	1,824 (27.0)	424 (24.8)	354 (21.2)	661 (32.0)	385 (29.1)	
No	4,940 (73.0)	1,284 (75.2)	1,313 (78.8)	1,404 (68.0)	939 (70.9)	
TG (mmol/L)	1.25 (1.00, 2.00)	1.00 (1.00, 1.45)	1.27 (0.91, 1.93)	1.00 (1.00, 2.00)	2.00 (1.39, 3.00)	<0.001
TC (mmol/L)	4.69 (4.00, 5.43)	4.00 (3.00, 4.93)	5.06 (4.41, 5.75)	4.00 (4.00, 5.00)	5.00 (4.62, 6.00)	<0.001
HDL-C (mmol/L)	1.25 (1.00, 1.69)	1.27 (1.00, 1.73)	1.56 (1.33, 1.80)	1.00 (1.00, 1.23)	1.21 (1.00, 1.60)	<0.001
LDL-C (mmol/L)	2.30 (2.00, 3.03)	2.43 (2.00, 3.00)	3.01 (2.50, 3.63)	2.00 (2.00, 3.00)	1.85 (1.00, 2.28)	<0.001

The difference in baseline characteristics of the included and excluded populations in this study are shown in Supplementary Table S1. In most baseline features, we found no statistically significant difference in the included and excluded groups. However, participants had a slightly higher rate of excessive drinking and a lower rate of inadequate cereal intake.

### 3.2. Association of RC concentration with increased risk of CVD outcomes

Among 6,764 individuals enrolled in the present study with a follow-up to 10 years (median 9.4 years), 199 participants had CVD, 160 had ASCVD, 171 had stroke, 37 had CHD, and 133 had IS. We explored the HRs for the incidence of various CVD outcomes based on the RC concentration. When RC was modeled as a categorical variable, individuals with a higher concentration of RC showed an increased risk of CVD, ASCVD, stroke, and IS. In the present study, for participants with RC >1.00 mmol/L vs. RC ≤0.25 mmol/L, the multivariable-adjusted results for CVD (HR, 1.79; 95% CI 1.15–2.79; *P-*value = 0.008), ASCVD (HR, 2.00; 95% CI 1.22–3.27; *P-*value = 0.007), stroke (HR, 1.66; 95% CI 1.02–2.69; *P-*value = 0.040), and IS (HR, 1.87, 95% CI 1.08–3.25; *P-*value = 0.034) were observed. No significant association between RC and CHD was found (HR, 2.11; 95% CI 0.83–5.37; *P-*value = 0.118) (Table 2).

**Table 2:** Incident risk of CVD outcomes associated with the baseline concentration of remnant cholesterol (categorical variable).

Outcomes	Remnant cholesterol (mmol/L)				
	Q1 (≤0.25)	Q2 (0.25–0.67)	Q3 (0.67–1.00)	Q4 (>1.00)	*P*-trend
CVD	40/1,708	33/1,667	77/2,065	49/1,324	
Model 1	1.00 (ref.)	1.10 (0.69–1.74)	**1.47 (1.00–2.16)** *	**1.85 (1.22–2.82)** **	**0** **.** **002**
Model 2	1.00 (ref.)	1.09 (0.68–1.74)	1.43 (0.98–2.10)	**1.77 (1.16–2.70)** **	**0** **.** **003**
Model 3	1.00 (ref.)	1.11 (0.69–1.78)	1.41 (0.95–2.08)	**1.79 (1.15–2.79)** **	**0** **.** **008**
ASCVD					
*n*/*N*	31/1,708	27/1,667	60/2,065	42/1,324	
Model 1	1.00 (ref.)	1.16 (0.69–1.94)	1.47 (0.96–2.27)	**2.04 (1.28–3.26)** **	**0** **.** **002**
Model 2	1.00 (ref.)	1.18 (0.70–2.00)	1.45 (0.94–2.24)	**1.99 (1.25–3.18)** **	**0** **.** **003**
Model 3	1.00 (ref.)	1.21 (0.71–2.07)	1.42 (0.91–2.21)	**2.00 (1.22–3.27)** **	**0** **.** **007**
Stroke					
*n*/*N*	34/1,708	31/1,667	66/2,065	40/1,324	
Model 1	1.00 (ref.)	1.20 (0.73–1.96)	1.48 (0.98–2.24)	**1.77 (1.12–2.81)** *	**0** **.** **008**
Model 2	1.00 (ref.)	1.22 (0.74–2.00)	1.45 (0.96–2.20)	**1.72 (1.09–2.73)** *	**0** **.** **014**
Model 3	1.00 (ref.)	1.27 (0.77–2.11)	1.38 (0.91–2.12)	**1.66 (1.02–2.69)** *	**0** **.** **040**
Ischemic stroke					
*n*/*N*	25/1,708	25/1,667	50/2,065	33/1,324	
Model 1	1.00 (ref.)	1.32 (0.75–2.30)	1.52 (0.94–2.46)	**1.99 (1.18–3.35)** **	**0** **.** **009**
Model 2	1.00 (ref.)	1.40 (0.79–2.46)	1.50 (0.93–2.44)	**1.98 (1.17–3.34)** *	**0** **.** **012**
Model 3	1.00 (ref.)	1.46 (0.82–2.61)	1.43 (0.87–2.34)	**1.87 (1.08–3.25)** *	**0** **.** **034**
CHD					
*n*/*N*	9/1,708	3/1,667	13/2,065	12/1,324	
Model 1	1.00 (ref.)	0.49 (0.13–1.69)	1.13 (0.48–2.65)	2.09 (0.88–4.97)	0.069
Model 2	1.00 (ref.)	0.44 (0.12–1.67)	1.05 (0.45–2.48)	1.87 (0.78–4.49)	0.103
Model 3	1.00 (ref.)	0.41 (0.11–1.57)	1.08 (0.45–2.60)	2.11 (0.83–5.37)	0.118

HR, hazards ratio; CI, confidence interval; CVD, cardiovascular disease; ASCVD, atherosclerotic cardiovascular disease; CHD, coronary heart disease.

Model 1: adjusted for age (continuous variable) and sex.

Model 2: Model 1 plus present smoking status, excessive drinking status, physical activity, insufficient cereal intake, insufficient vegetable and fruit intake, bean product intake, animal entrail intake, and excessive meat intake.

Model 3: Model 2 plus BMI (continuous variable), lipid-lowering drug use, LDL-C (continuous variable), diabetes, and hypertension.

* *P *< 0.05.

** *P *< 0.01.

Bold values represent statistical significance.

When RC was modeled as a continuous variable, participants had a significantly increased risk of CVD by 24.6% (HR, 1.25, 95% CI 1.02–1.52) and ASCVD by 28.0% (HR, 1.28, 95% CI 1.03–1.56) after fully adjusting for the models. Significantly higher risks for stroke (HR, 1.23; 95% CI 1.00–1.49), IS (HR, 1.26; 95% CI 1.01–1.56), and CHD (HR, 1.49; 95% CI 1.00–2.22) were found for participants after adjusting for Model 2 covariates, but the significance vanished in Model 3 (Table 3).

**Table 3:** Incident risk of CVD outcomes associated with the baseline concentration of remnant cholesterol (continuous variable).

Outcomes	HR (95% CI)			
	Events	Model 1	Model 2	Model 3
CVD	199/6,764	**1.28** (1.07–1.53)**	**1.26** (1.06–1.51)*	**1.25** (1.02–1.52)*
ASCVD	160/6,764	**1.31** (1.08–1.59)**	**1.30** (1.07–1.58)**	**1.28** (1.03–1.59)*
Stroke	171/6,764	**1.24** (1.02–1.50)*	**1.23** (1.01–1.49)*	1.18 (0.96–1.47)
Ischemic stroke	133/6,764	**1.27** (1.02–1.57)*	**1.26** (1.01–1.56)*	1.21 (0.95–1.54)
CHD	37/6,764	**1.51** (1.03–2.22)*	**1.49** (1.00–2.22)*	1.52 (0.98–2.35)

HR, hazards ratio; CI, confidence interval; CVD, cardiovascular disease; ASCVD, atherosclerotic cardiovascular disease; CHD, coronary heart disease.

Model 1: adjusted for age (continuous variable) and sex.

Model 2: Model 1 plus present smoking status, excessive drinking status, physical activity, insufficient cereal intake, insufficient vegetable and fruit intake, bean product intake, animal entrail intake, and excessive meat intake.

Model 3: Model 2 plus BMI (continuous variable), lipid-lowering drug use, LDL-C (continuous variable), diabetes, and hypertension.

* *P *< 0.05.

** *P *< 0.01.

Bold values represent statistical significance.

### 3.3. Dose–response association between RC and cardiovascular outcomes

**Figure 2:**
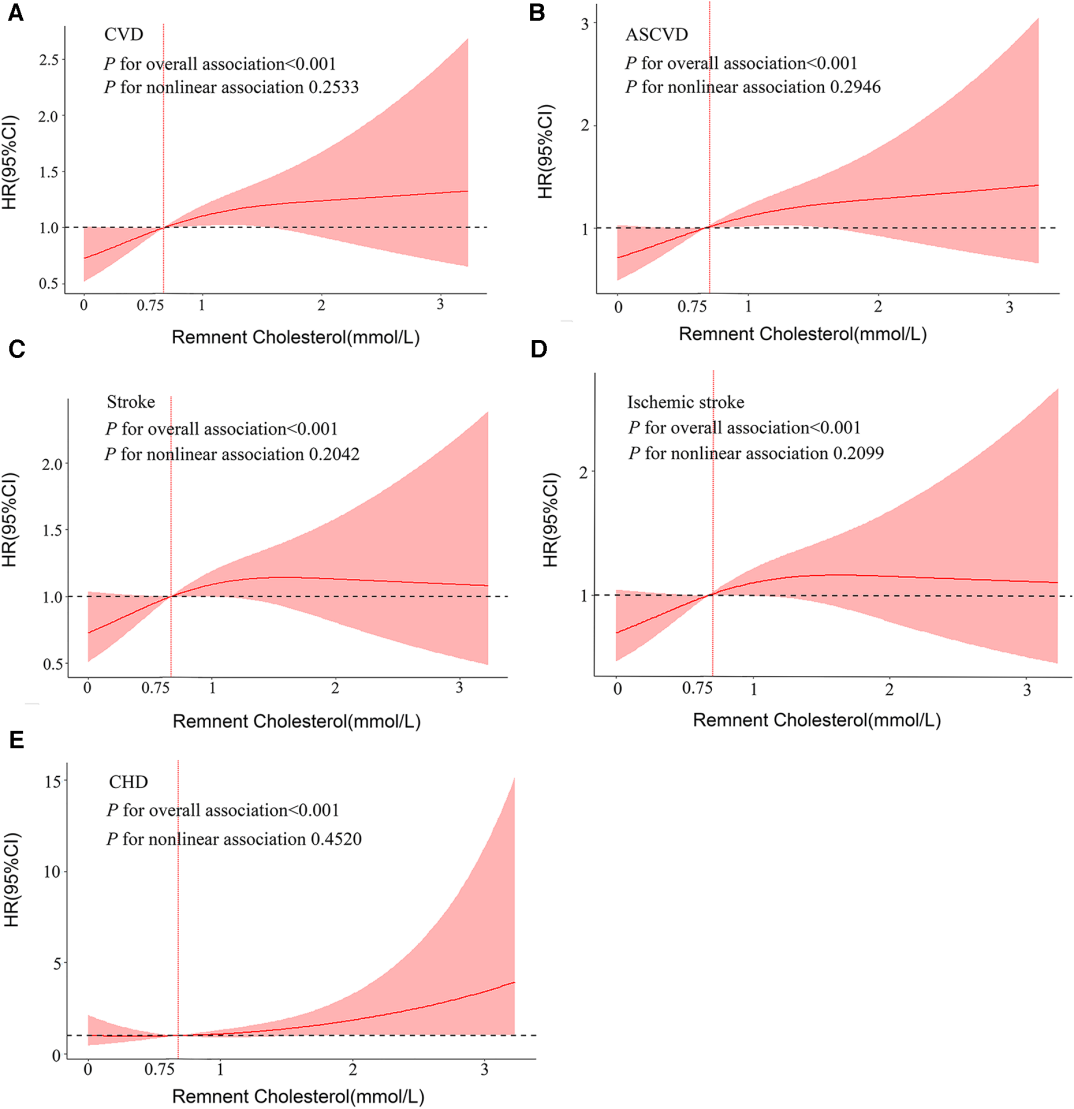
Dose–response associations between RC and cardiovascular outcomes. Restricted cubic splines display the HR of cardiovascular outcomes with 95% confidence intervals according to the concentration of RC. All analyses were adjusted for Model 3 covariates. HR, hazards ratio; CI, confidence interval; CVD, cardiovascular disease; ASCVD, atherosclerotic cardiovascular disease; CHD, coronary heart disease.

After fully adjusting for the models, RCS regression presented significant overall associations of RC with CVD (*P*-overall <0.001), ASCVD (*P*-overall <0.001), stroke (*P*-overall <0.001), IS (*P*-overall <0.001), and CHD (*P*-overall <0.001) (Figure 2). More importantly, when RC concentration was >0.75 mmol/L, the risk of CVD dramatically increased.

### 3.4. Subgroup analysis

**Figure 3:**
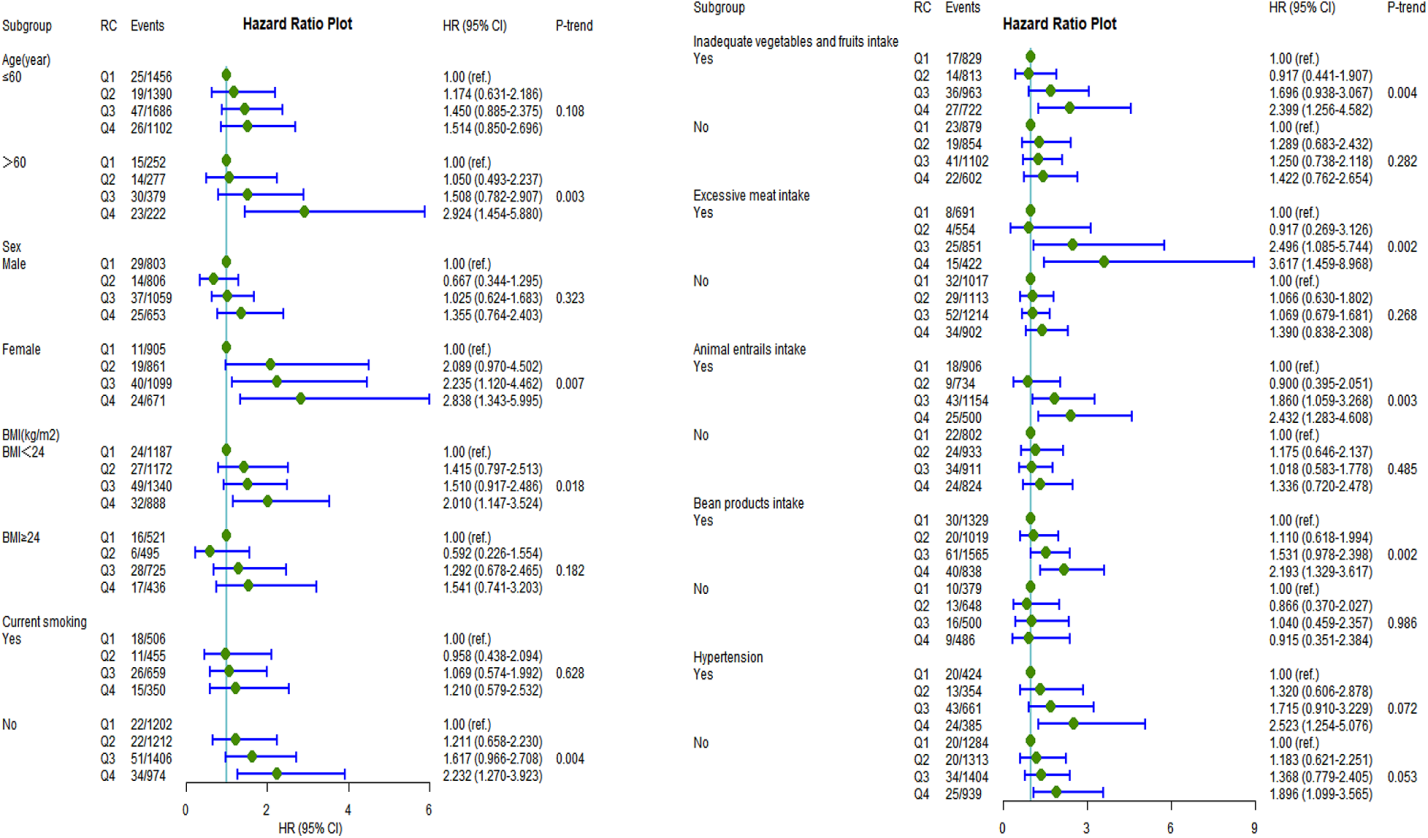
Incident risk of CVD associated with remnant cholesterol by age, sex, BMI, current smoking, inadequate vegetable and fruit intake, excessive meat intake, bean product intake, animal entrail intake, and hypertension (yes or no). All analyses were adjusted for Model 3 covariates. HR, hazards ratio; CI, confidence interval; CVD, cardiovascular disease; BMI, body mass index.

After fully adjusting for potential confounders, individuals in the highest RC quartile had a significantly higher incidence of CVD than those in the lowest RC quartile in the following subgroups (Figure 3): women (HR: 2.84, 95% CI: 1.34–6.00; *P*value = 0.007), aged >60 years (HR: 2.92, 95% CI:1.45–5.88; *P-*value = 0.003), noncurrent smokers (HR: 2.23, 95% CI 1.27–3.92; *P-*value = 0.004), BMI <24 kg/m^2^ (HR: 2.01, 95% CI: 1.15–3.52; *P-*value = 0.018), inadequate vegetable and fruit intake (HR: 2.40, 95% CI: 1.26–4.58; *P-*value = 0.004), excessive meat intake (HR: 3.62, 95% CI: 1.46–8.97; *P-*value = 0.002), animal entrail intake (HR: 2.43, 95% CI: 1.28–4.61; *P-*value = 0.003), and bean product intake (HR: 2.19, 95% CI: 1.33–3.17; *P-*value = 0.002). The same trend was found for ASCVD, stroke, and IS prevalence among the following subgroups: aged >60 years, women, noncurrent smokers, insufficient vegetable and fruit intake, excessive meat intake, and animal entrail intake (Supplementary Figures S1–S3).

### 3.5. Sensitivity analysis

Moreover, participants who took lipid-lowering drugs at baseline were excluded for sensitive analysis. The relationship between RC and the risks of CVD outcomes did not markedly change. Individuals with higher RC concentrations still showed a higher risk for CVD, ASCVD, stroke, and IS (Supplementary Table S2, Model 3). When RC was regarded as a continuous variable, the CVD (HR, 1.24; 95% CI 1.02–1.51) and ASCVD (HR, 1.26; 95% CI 1.02–1.57) risks remained increased (Supplementary Tables S2–S3, Model 3).

After adequate adjustment for confounders (including HDL-C), the association between RC and CVD remained robust (Supplementary Tables S4–S5, Model 3), and the results remained robust after including diabetic nephropathy in the adjusted model (Supplementary Tables S6–S7, Model 3).

## 4. Discussion

This is the first large-scale prospective cohort study to investigate the association between RC and the incidence of CVD outcomes based on the representative population in southwest China. The Cox fully adjusted model indicated a roughly twofold increased risk for CVD, ASCVD, and IS with elevated RC concentration. This relationship remained significant even after adjusting for LDL-C, indicating that the correlation between RC and incidence of CVD outcomes was independent of LDL-C. Importantly, our study found that the increasing trend of CVD risk began when RC ≥0.75 mmol/L. Furthermore, a stronger association was observed among participants aged >60 years, women, hypertension patients, and participants with unhealthy diet patterns.

It has been demonstrated through several observational studies and Mendelian randomization experiments that RC is crucial to the pathophysiology of CVD. A randomized controlled trial at the Spanish PREDIMED Research Center suggested that the decreased triglycerides (which can be used as a marker of RC) caused by icosapent ethyl, a highly purified omega-3 fatty acid, can contribute to a 25% reduction in the risk of ASCVD (27), which is consistent with our findings. Similar results were found in the US and Copenhagen cohort populations by Joshi and Duran et al. (13, 15, 28). Notably, the relationship of RC with CVD risk was independent of LDL-C in the above studies. These results imply that proper management of RC could provide cardiovascular benefits. However, in current clinical practice, most clinicians use statins to reduce LDL-C concentrations without considering residual CVD risk (11). Although there is no consensus on whether treating high levels of RC could prevent cardiovascular disease, some consensus statements and guidelines recommend such treatment as a priority strategy (29). There is a large amount of new research studying new treatments focusing on statins, PcsK9 inhibitors, fibrates, and omega-3 fatty acids (OM3FAs) for elevated RC. Although beta-blockers and fish oils have lowered RC, they have not been consistently successful in reducing CVD. For targeted drug therapy, genetic studies are likewise being vigorously pursued. For instance, angiopoietin–like protein 3 (ANGPTL3), apolipoprotein C3 (APOC3), angiopoietin-like protein 4 (ANGPTL4). APOC3 and ANGPTL3 have been targeted for inhibition by antibody, antisense RNA, or RNAi approaches. Inhibition of any of these molecules decreases RC but increases or lowers HDL levels (30). Although no precisely targeted therapies are available, many novel agents in late-stage research have been developed. Large clinical trials of such drugs in patients with high cardiovascular risk and elevated RC levels are urgently needed to demonstrate the effectiveness of these approaches. Based on the large-scale prospective study conducted in a representative Southwest Chinese general population, our results further support that RC might as a potential preventive/therapeutic target for the CVD susceptibility population, especially those with RC concentrations greater than 0.75 mmol/L. Consistently, in the PREDIMED elderly subjects and Northeast Rural Cardiovascular Health Study, CVD risk increased when RC concentrations were greater than 0.78 mmol/L and 0.84 mmol/L, respectively (14, 18). Importantly, our study covered both urban and rural representative populations in southwest China; thus, our results were more generalizable than previous studies.

TRL-transformed lipoprotein remnants are vital for the development and progression of atherosclerosis (31). Compared with LDL, RC may be preferentially retained in the intima due to its larger size and higher cholesterol content (29, 32). Once RC enters the arterial wall, it is subsequently directly phagocyted by the macrophage, making it more likely to be retained in the endarterium and eventually lead to the formation of atherosclerotic plaques (33, 34). In addition, RC derived from the hydrolysis of TRLs induces the production of IL-8, IL-1, cytokines (TNF-a), and proatherogenic adhesion molecules (35). The activated coagulation cascade via prothrombinase and excessive inflammation also contributes to increased CVD risk (36, 37). Taken together, all the above aberrant processes elicited by RC well-orchestrated lead to the occurrence of CVD.

In the subgroup analysis, the adverse correlation between RC and CVD outcomes in women, participants aged >60 years, and participants with BMI <24 kg/m^2^ was stronger, which is partially controversial with previous studies (13, 14). Compared to men, this may be related to the lack of estrogen in older women and unfavorable lipid changes after menopause (38). It is noteworthy that compared to Western populations, the Chinese have a smaller body size and lower BMI but higher visceral fat and a lower fat-free mass (39). This race difference contributes to more abnormal metabolic at the same BMI in China compared with people from other countries (40). Thus, our results suggest that Chinese people with BMI <24 kg/m^2^ and abnormal metabolism should be paid more attention. More importantly, our study observed a stronger association among those with low vegetable and fruit intake, excessive meat intake, and consumption of animal entrails. This is consistent with prior research showing that a healthy diet (such as the Mediterranean diet) could reduce the risk of CVD.

### 4.1. Advantages and limitations

This study, to our knowledge, is the first sizeable longitudinal cohort sample based on the Southwest Chinese general population to assess the relationship between RC and CVD and its subtypes. Moreover, we first adjusted for dietary habits in the multivariable model. Notably, we calculated the cutoff values for RC's increased susceptibility to CVD, which may facilitate clinical practice or early screening of people at risk for CVD. However, several limitations should also be acknowledged. First, we calculated the RC using Friedewald's formula instead of directly measuring the RC. Second, even if most confounding factors were adjusted, unmeasured or unidentified factors such as GFR, urine albumin-to-creatinine ratio (UACR), and fetal-liver infusion (FIL) may have potential confounding effects. Third, because our participants were enrolled from Southwest China, caution must be made when extrapolating our result to other nationwide populations. Fourth, due to the observational nature, the causality of RC on CVD risk should be supported in additional randomized controlled trial studies. Fifth, we failed to consider how lipid profiles vary over time in this investigation.

## 5. Conclusions

In this large-scale prospective cohort study based on the Southwest China general population, we found that a high level of RC is a risk factor for developing CVD, independent of LDL-C. RC may serve as an important and new intervention target biomarker to reduce CVD, especially for a sensitive subject whose RC level is >0.75 mmol/L. More importantly, the importance of RC, which could be one low-cost and wide-availability lipid biochemical biomarker, must be considered. We believe that combining traditional influencing factors with new lipid markers constitutes a predictive model or screening strategy that may improve detection and early intervention in high-risk populations and reduce cardiovascular detection rates and the incidence of poor prognosis.

## Data Availability

The raw data supporting the conclusions of this article will be made available by the authors without undue reservation.
