# Supplementary material for: Association of remnant cholesterol with CVD incidence: a general population cohort study in Southwest China
Source: Front Cardiovasc Med. 2023 Nov 27;10:1286286. doi: 10.3389/fcvm.2023.1286286 (PMC10711629; doi:10.3389/fcvm.2023.1286286)
Supplement: Supplementary file 1 [file Datasheet1.pdf]

**supplementary materials for**

# Association of remnant cholesterol with CVD incidence: a general population cohort study in Southwest China

**Chengxi Liu[1,2*], Mi Dai[3*], Kunming Tian[2,4], Shiyu Zhou[4], Lei Luo[2], Zhiying Zeng[1]， Xuelian Yan[3], Ying Xiao[3], Yiying Wang[5], Renli Deng[4], Xiuhong Lei[1#],Tao Liu[5#]**

**[1]Department of Anesthesiology, The Second Affiliated Hospital, Hengyang Medical School, University of South China, Hengyang, China**

**[2]Department of Preventive Medicine, School of Public Health, Zunyi Medical University, Zunyi, China**

**[3]The Third Affiliated Hospital of Zunyi Medical University(The First People's Hospital of Zunyi), Zunyi, China**

**[4]Department of Nursing, Affiliated Hospital of Zunyi Medical University, Zunyi, China**

**[5]Department of Chronic Disease Prevention and Control, Guizhou Disease Prevention and Control, Guiyang, China**

**[*] Chengxi Liu and Mi Dai contributed equally to this work.**

**#Corresponding author:**

**Tao Liu, liutao9099No_2@163.com, Department of Chronic Disease Prevention and Control, Guizhou Disease Prevention and Control, Guiyang, Guizhou, China**

**Xiuhong Lei, Master, Department of Anesthesiology, The Second Affiliated Hospital, Hengyang Medical School, University of South China, Hengyang City, Hunan Province, China; E-mail: leixiuhong@126.com**

**Supplementary Table 1.** The comparison of baseline characteristics between the included and excluded populations.

**Supplementary Table 2.** Excluding subjects with lipid-lowering drugs used at baseline, the incident risk of CVD Outcomes associated with baseline concentration of RC (categorical variable).

**Supplementary Table 3.** Excluding subjects with lipid-lowering drugs used at baseline, the incident risk of CVD Outcomes associated with baseline concentration of RC (continuous variable).

**Supplementary Table 4.** The incident risk of CVD Outcomes associated with baseline concentration of Remnant Cholesterol (categorical variable).

**Supplementary Table 5.** The incident risk of CVD Outcomes associated with baseline concentration of Remnant Cholesterol (continuous variable).

**Supplementary Table 6.** The incident risk of CVD Outcomes associated with baseline concentration of Remnant Cholesterol (categorical variable).

**Supplementary Table 7.** The incident risk of CVD Outcomes associated with baseline concentration of Remnant Cholesterol (continuous

variable).

**Supplementary Figure 1.** RC in relation to ASCVD for different subgroups.

**Supplementary Figure 2.** RC in relation to Stroke for different subgroups.

**Supplementary Figure 3.** RC in relation to IS for different subgroups.

Table S1. The comparison of baseline characteristics between the included and excluded populations.

| Characteristics | Included subjects N=6764 | Excluded subjects N=2516 | *P* |
|---|---|---|---|
| Age (years) | 44.81±15.09 | 44.69±17.38 | 0.742 |
| BMI (kg/m2) | 22.85±3.26 | 22.76±3.57 | 0.303 |
| Sex n (%) | | | 0.674 |
| Male | 3228 (47.7%) | 1214(48.2%) | |
| Female | 3536 (52.3%) | 1303 (51.8%) | |
| Current smoking n (%) | | | 0.621 |
| Yes | 1970 (29.1%) | 720 (28.6%) | |
| No | 4794 (70.9%) | 1797 (71.4%) | |
| Excessive drinking n (%) | | | **0.040** |
| Yes | 722 (10.7%) | 232 (9.2%) | |
| No | 6042 (89.3%) | 2285 (90.8%) | |
| Physical activity | | | 0.159 |
| Never | 6215 (91.9%) | 2282 (90.8%) | |
| 1-2 days per week | 139 (2.1%) | 66 (2.6%) | |
| ≥3 days per week | 410 (6.0%) | 165 (6.6%) | |
| Inadequate cereal intake | | | **＜0.001** |
| Yes | 1108 (16.4%) | 543 (21.6%) | |
| No | 5656 (83.6%) | 1974 (78.4%) | |
| Inadequate vegetables and fruits intake | | | 0.113 |
| Yes | 3437 (51.0%) | 1326 (52.9%) | |
| No | 3297 (49.0%) | 1181 (47.1%) | |
| Excessive meat intake | | | 0.787 |
| Yes | 1835 (27.1%) | 690 (27.4%) | |
| No | 4929 (72.9%) | 1827 (72.6%) | |

| | | | |
|---|---|---|---|
| Bean products intake | | | 0.771 |
| Yes | 4752 (70.3%) | 1775 (70.6%） | |
| No | 2012 (29.7%) | 740 (29.4%) | |
| Animal entrails intake | | | 0.095 |
| Yes | 3297 (48.7%) | 1173 (46.8%) | |
| No | 3467 (51.3%) | 1334 (53.2%) | |
| diabetes | | | 0.881 |
| Yes | 523 (7.7%) | 197 (7.8%) | |
| No | 6241 (92.3%) | 2320 (92.2%) | |
| hypertension | | | 0.051 |
| Yes | 1918 (28.4%) | 660 (26.2%) | |
| No | 4846 (71.6%) | 1857 (73.8%) | |
| Lipid-lowering drugs use | | | 0.768 |
| Yes | 6706 (99.1%) | 2497 (99.2%) | |
| No | 58 (0.9%) | 20 (0.8%) | |

BMI: body mass index. Data are expressed as mean ± SD or as *n* (%).

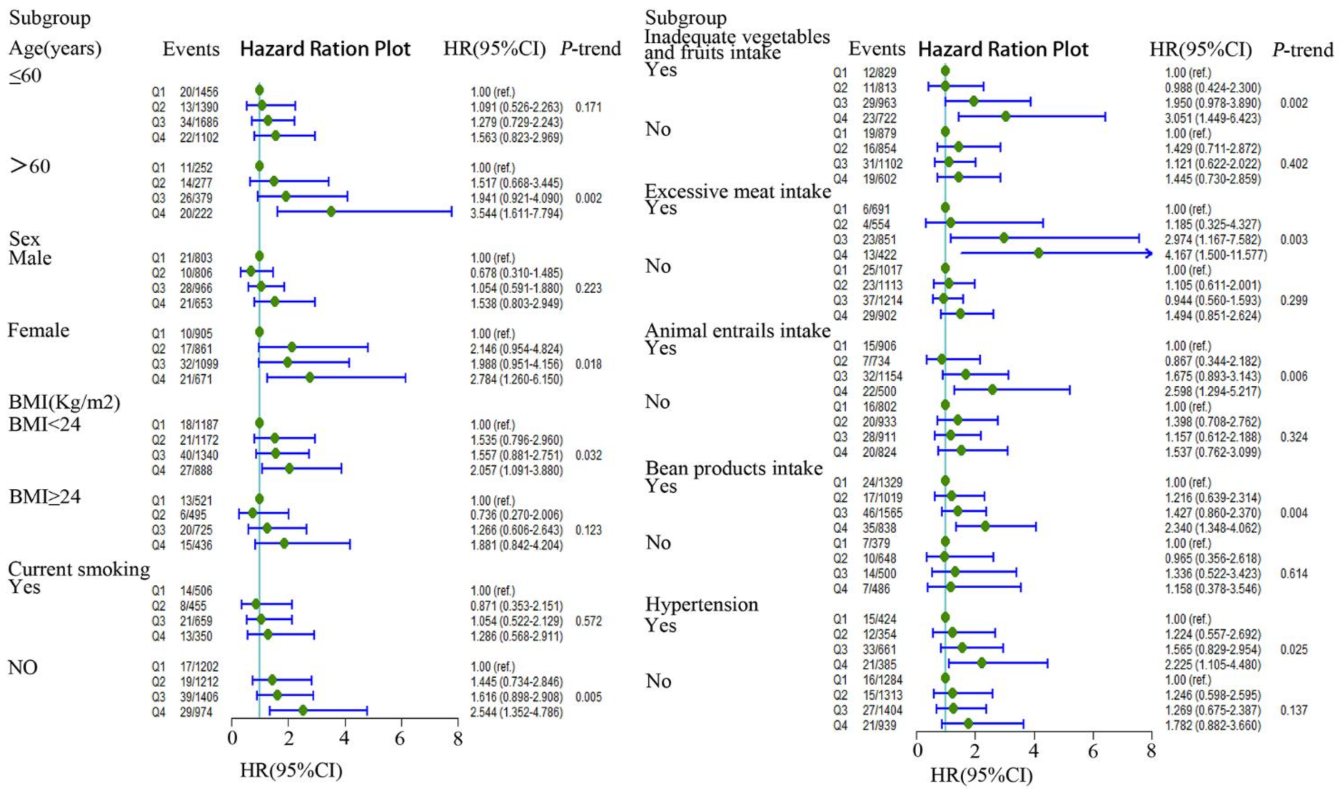


Figure S1. The incident risk of ASCVD is associated with Remnant Cholesterol by age, sex, BMI, current smoking, Inadequate vegetables and fruits intake, Excessive meat intake, Bean products intake, Animal entrails intake, and hypertension (yes or no). All analyses were adjusted for model 3 covariates. Abbreviations: HR, hazard ratios; CI, confidence interval; ASCVD, Atherosclerotic cardiovascular disease; BMI, body mass index.

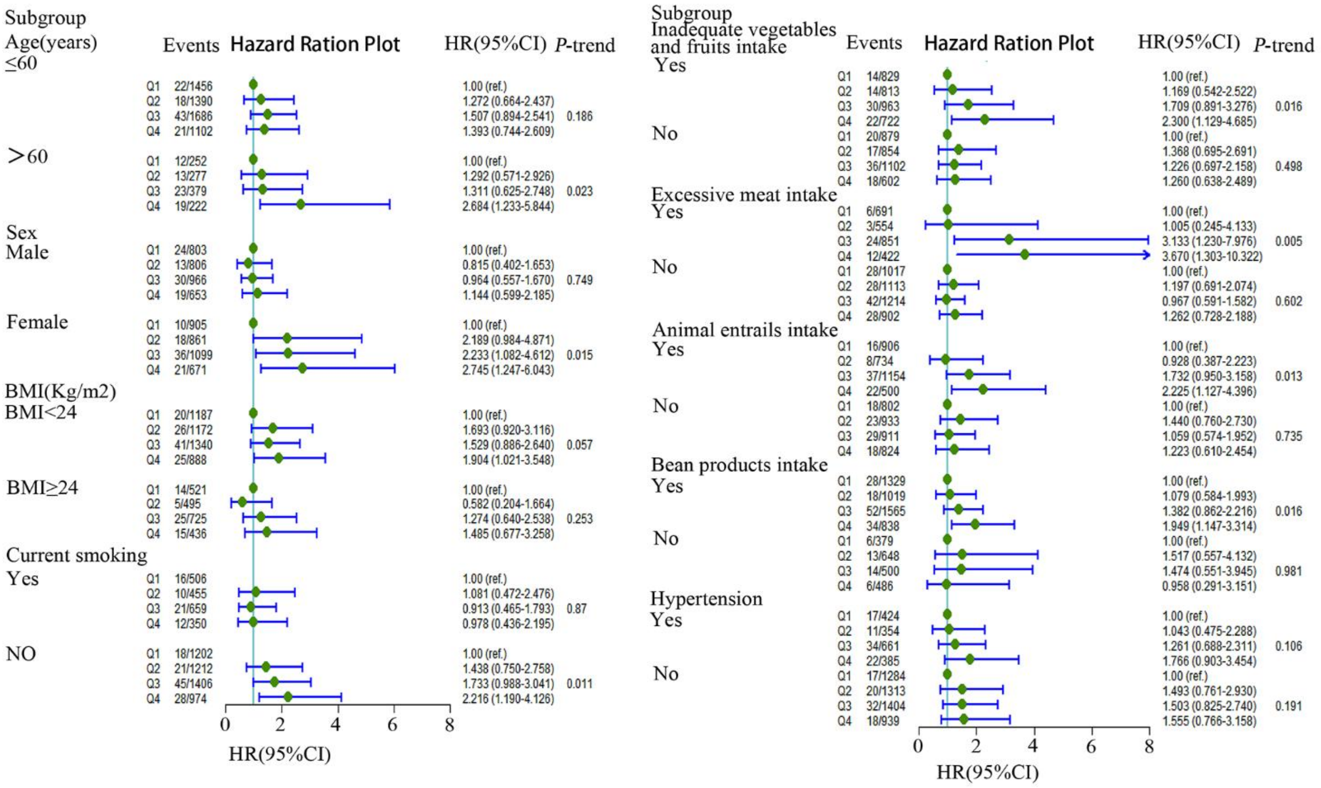


Figure S2. The incident risk of Stroke is associated with Remnant Cholesterol by age, sex, BMI, current smoking, Inadequate vegetables and fruits intake, Excessive meat intake, Bean products intake, Animal entrails intake, and hypertension (yes or no). All analyses were adjusted for model 3 covariates. Abbreviations: HR, hazard ratios; CI, confidence interval; BMI, body mass index.

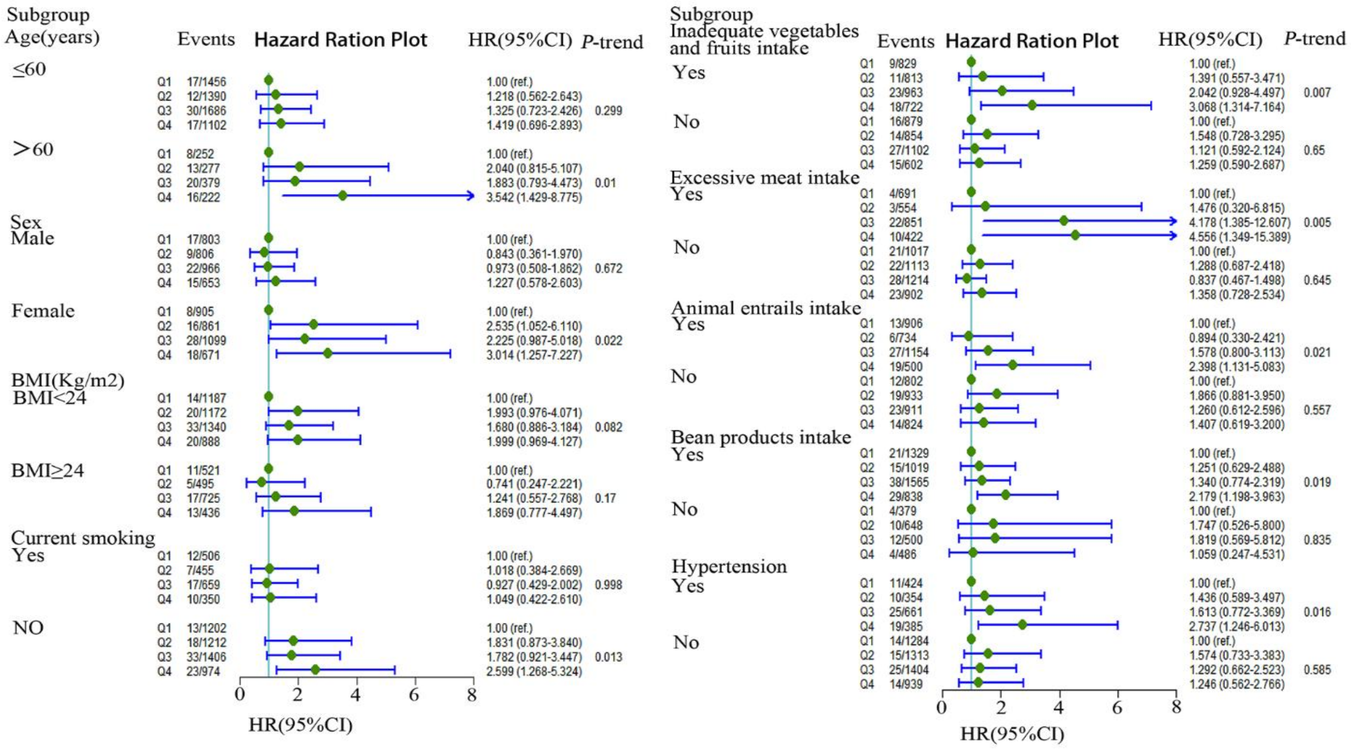


Figure S3. The incident risk of Ischemic stroke is associated with Remnant Cholesterol by age, sex, BMI, current smoking, Inadequate vegetables and fruits intake, Excessive meat intake, Bean products intake, Animal entrails intake, and hypertension (yes or no). All analyses were adjusted for model 3 covariates. Abbreviations: HR, hazard ratios; CI, confidence interval; BMI, body mass index.

Table S2. Excluding subjects with lipid-lowering drugs used at baseline, the incident risk of CVD Outcomes associated with baseline concentration of RC (categorical variable).

| **Outcomes** | Remnant Cholesterol(mmol/L) | | | | |
|---|---|---|---|---|---|
| | Q1 (≤0.25） | Q2 (0.25-0.67) | Q3 (0.67-1.00） | Q4 (＞1.00） | *P*-trend |
| **CVD** | 40/1696 | 33/1659 | 73/2045 | 47/1306 | |
| Model 1 | 1.00 (ref.) | 1.10 (0.69-1.74) | 1.41 (0.96-2.08) | **1.81 (1.18-2.76)**** | **0.003** |
| Model 2 | 1.00 (ref.) | 1.08 (0.68-1.73) | 1.38 (0.93-2.02) | **1.71 (1.12-2.62)*** | **0.007** |
| Model 3 | 1.00 (ref.) | 1.09 (0.68-1.76) | 1.37 (0.92-2.04) | **1.78 (1.14-2.78)*** | **0.010** |
| **ASCVD** | | | | | |
| n/N | 31/1696 | 27/1659 | 57/2045 | 40/1306 | |
| Model 1 | 1.00 (ref.) | 1.16 (0.69-1.94) | 1.42 (0.91-2.19) | **1.98 (1.24-3.18)**** | **0.004** |
| Model 2 | 1.00 (ref.) | 1.18 (0.70-2.00) | 1.39 (0.90-2.16) | **1.92 (1.19-3.08)**** | **0.006** |
| Model 3 | 1.00 (ref.) | 1.21 (0.71-2.06) | 1.38 (0.88-2.16) | **1.96 (1.20-3.23)**** | **0.010** |
| **Stroke** | | | | | |
| n/N | 34/1696 | 31/1659 | 62/2045 | 38/1306 | |
| Model 1 | 1.00 (ref.) | 1.20 (0.73-1.95) | 1.41 (0.93-2.14) | **1.71 (1.08-2.72)*** | **0.017** |
| Model 2 | 1.00 (ref.) | 1.21 (0.74-1.99) | 1.38 (0.91-2.10) | **1.65 (1.03-2.63)*** | **0.029** |
| Model 3 | 1.00 (ref.) | 1.26 (0.76-2.08) | 1.34 (0.87-2.06) | **1.64 (1.01-2.68)*** | **0.050** |
| **Ischaemic stroke** | | | | | |

| | | | | | |
|---|---|---|---|---|---|
| n/N | 25/1696 | 25/1659 | 47/2045 | 31/1306 | |
| Model 1 | 1.00 (ref.) | 1.31 (0.75-2.30) | 1.44 (0.89-2.35) | **1.90 (1.12-3.22)*** | **0.018** |
| Model 2 | 1.00 (ref.) | 1.39 (0.79-2.45) | 1.43 (0.88-2.32) | **1.88 (1.10-3.20)*** | **0.026** |
| Model 3 | 1.00 (ref.) | 1.46 (0.82-2.60) | 1.37 (0.84-2.26) | **1.83 (1.05-3.20)*** | **0.049** |
| CHD | | | | | |
| n/N | 9/1696 | 3/1659 | 13/2045 | 11/1306 | |
| Model 1 | 1.00 (ref.) | 0.48 (0.13-1.80) | 1.14 (0.49-2.67) | 1.94 (0.80-4.71) | 0.099 |
| Model 2 | 1.00 (ref.) | 0.44 (0.12-1.64) | 1.06 (0.45-2.50) | 1.70 (0.70-4.16) | 0.154 |
| Model 3 | 1.00 (ref.) | 0.40 (0.10-1.51) | 1.13 (0.47-2.71) | 2.01 (0.78-5.17) | 0.137 |

Abbreviations: HR, hazard ratios; CI, confidence interval.
CVD, cardiovascular disease; ASCVD, atherosclerotic cardiovascular disease; CHD, coronary heart disease.
Model 1: adjusted for age(continuous variable), sex
Model 2: model 1 plus present smoking status, excessive drinking status, physical activity, cereal intake, vegetables and fruits intake, bean products intake, Animal entrails intake, and excessive meat intake.
Model 3: model 2 plus BMI(continuous variable), Lipid-lowering drugs use, LDL-C(continuous variable), diabetes, hypertension.
**P*<0.05;***P*<0.01

Table S3. Excluding subjects with lipid-lowering drugs used at baseline, the incident risk of CVD Outcomes associated with baseline concentration of RC (continuous variable).

| RC continuous | HR (95% CI) | | | |
|---|---|---|---|---|
| | Events | Model 1 | Model 2 | Model 3 |
| CVD | 193/6706 | **1.26 (1.05-1.51)*** | **1.24 (1.04-1.50)*** | **1.24 (1.02-1.51)*** |
| ASCVD | 155/6706 | **1.29 (1.06-1.57)*** | **1.27 (1.04-1.56)*** | **1.26 (1.02-1.57)*** |
| Stroke | 165/6706 | 1.22 (0.99-1.48) | 1.20 (0.98-1.47) | 1.18 (0.95-1.46) |
| Ischemic stroke | 128/6706 | 1.24 (0.99-1.55) | 1.23 (0.98-1.53) | 1.19 (0.94-1.53) |
| MI | 36/6706 | 1.46 (0.98-2.17) | 1.43 (0.95-2.15) | 1.49 (0.96-2.32) |

Abbreviations: HR, hazard ratios; CI, confidence interval.

CVD, cardiovascular disease; ASCVD, atherosclerotic cardiovascular disease; CHD, coronary heart disease.

Model 1: adjusted for age(continuous variable), sex

Model 2: model 1 plus present smoking status, excessive drinking status, physical activity, cereal intake, vegetables and fruits intake, bean products intake, Animal entrails intake, and excessive meat intake.

Model 3: model 2 plus BMI(continuous variable), Lipid-lowering drugs use, LDL-C(continuous variable), diabetes, hypertension.

*$P$<0.05;**$P$<0.01

Table S4 The incident risk of CVD Outcomes associated with baseline concentration of Remnant Cholesterol (categorical variable).

| Outcomes | Remnant Cholesterol(mmol/L) | | | | |
|---|---|---|---|---|---|
| | Q1 (≤0.25） | Q2 (0.25-0.67) | Q3 (0.67-1.00） | Q4 (＞1.00） | *P*-trend |
| CVD | 40/1708 | 33/1667 | 77/2065 | 49/1324 | |
| Model 1 | 1.00 (ref.) | 1.10 (0.69-1.74) | **1.47 (1.00-2.16)*** | **1.85 (1.22-2.82)**** | **0.002** |
| Model 2 | 1.00 (ref.) | 1.09 (0.68-1.74) | 1.43 (0.98-2.10) | **1.77 (1.16-2.70)**** | **0.003** |
| Model 3 | 1.00 (ref.) | 1.11 (0.69-1.80) | 1.40 (0.94-2.08) | **1.79 (1.15-2.77)**** | **0.008** |
| ASCVD | | | | | |
| n/N | 31/1708 | 27/1667 | 60/2065 | 42/1324 | |
| Model 1 | 1.00 (ref.) | 1.16 (0.69-1.94) | 1.47 (0.96-2.27) | **2.04 (1.28-3.26)**** | **0.002** |
| Model 2 | 1.00 (ref.) | 1.18 (0.70-2.00) | 1.45 (0.94-2.24) | **1.99 (1.25-3.18)**** | **0.003** |
| Model 3 | 1.00 (ref.) | 1.22 (0.72-2.10) | 1.40 (0.89-2.20) | **2.00 (1.22-3.27)**** | **0.008** |
| Stroke | | | | | |
| n/N | 34/1708 | 31/1667 | 66/2065 | 40/1324 | |
| Model 1 | 1.00 (ref.) | 1.20 (0.73-1.96) | 1.48 (0.98-2.24) | **1.77 (1.12-2.81)*** | **0.008** |
| Model 2 | 1.00 (ref.) | 1.22 (0.74-2.00) | 1.45 (0.96-2.20) | **1.72 (1.09-2.73)*** | **0.014** |
| Model 3 | 1.00 (ref.) | 1.27 (0.76-2.11) | 1.39 (0.90-2.13) | **1.66 (1.02-2.69)*** | **0.040** |
| Ischaemic stroke | | | | | |
| n/N | 25/1708 | 25/1667 | 50/2065 | 33/1324 | |
| Model 1 | 1.00 (ref.) | 1.32 (0.75-2.30) | 1.52 (0.94-2.46) | **1.99 (1.18-3.35)**** | **0.009** |
| Model 2 | 1.00 (ref.) | 1.40 (0.79-2.46) | 1.50 (0.93-2.44) | **1.98 (1.17-3.34)*** | **0.012** |

| | | | | | |
|---|---|---|---|---|---|
| Model 3 | 1.00 (ref.) | 1.46 (0.82-2.61) | 1.44 (0.87-2.36) | **1.88 (1.08-3.25)*** | **0.033** |
| CHD | | | | | |
| n/N | 9/1708 | 3/1667 | 13/2065 | 12/1324 | |
| Model 1 | 1.00 (ref.) | 0.49 (0.13-1.69) | 1.13 (0.48-2.65) | 2.09 (0.88-4.97) | 0.069 |
| Model 2 | 1.00 (ref.) | 0.44 (0.12-1.67) | 1.05 (0.45-2.48) | 1.87 (0.78-4.49) | 0.103 |
| Model 3 | 1.00 (ref.) | 0.44 (0.12-1.70) | 0.98 (0.40-2.39) | 2.10 (0.83-5.30) | 0.143 |

Abbreviations: HR, hazard ratios; CI, confidence interval.
CVD, cardiovascular disease; ASCVD, atherosclerotic cardiovascular disease; CHD, coronary heart disease.
Model 1: adjusted for age(continuous variable), sex
Model 2: model 1 plus present smoking status, excessive drinking status, physical activity, cereal intake, vegetables and fruits intake, bean products intake, Animal entrails intake, and excessive meat intake.
Model 3: model 2 plus BMI(continuous variable), Lipid-lowering drugs use, LDL-C(continuous variable), HDL-C(continuous variable), diabetes, hypertension.
**P*<0.05;***P*<0.01

Table S5 The incident risk of CVD Outcomes associated with baseline concentration of Remnant Cholesterol (continuous variable).

| Outcomes | HR (95% CI) | | | |
| --- | --- | --- | --- | --- |
| | Events | Model 1 | Model 2 | Model 3 |
| CVD | 199/6764 | **1.28 (1.07-1.53)**** | **1.26 (1.06-1.51)*** | **1.23 (1.01-1.50)*** |
| ASCVD | 160/6764 | **1.31 (1.08-1.59)**** | **1.30 (1.07-1.58)**** | **1.26 (1.02-1.57)*** |
| Stroke | 171/6764 | **1.24 (1.02-1.50)*** | **1.23 (1.01-1.49)*** | 1.18 (0.95-1.46) |
| Ischemic stroke | 133/6764 | **1.27 (1.02-1.57)*** | **1.26 (1.01-1.56)*** | 1.21 (0.95-1.53) |
| CHD | 37/6764 | **1.51 (1.03-2.22)*** | **1.49 (1.00-2.22)*** | 1.47 (0.94-2.29) |

Abbreviations: HR, hazard ratios; CI, confidence interval.

CVD, cardiovascular disease; ASCVD, atherosclerotic cardiovascular disease; CHD, coronary heart disease.

Model 1: adjusted for age(continuous variable), sex

Model 2: model 1 plus present smoking status, excessive drinking status, physical activity, cereal intake, vegetables and fruits intake, bean products intake, Animal entrails intake, and excessive meat intake.

Model 3: model 2 plus BMI(continuous variable), Lipid-lowering drugs use, LDL-C(continuous variable), HDL-C(continuous variable), diabetes, hypertension.

**P*<0.05;***P*<0.01

Table S6 The incident risk of CVD Outcomes associated with baseline concentration of Remnant Cholesterol (categorical variable).

| Outcomes | Remnant Cholesterol(mmol/L) | | | | |
|---|---|---|---|---|---|
| | Q1 (≤0.25) | Q2 (0.25-0.67) | Q3 (0.67-1.00) | Q4 (＞1.00) | *P*-trend |
| CVD | 40/1708 | 33/1667 | 77/2065 | 49/1324 | |
| Model 1 | 1.00 (ref.) | 1.10 (0.69-1.74) | **1.47 (1.00-2.16)*** | **1.85 (1.22-2.82)**** | **0.002** |
| Model 2 | 1.00 (ref.) | 1.09 (0.68-1.74) | 1.43 (0.98-2.10) | **1.77 (1.16-2.70)**** | **0.003** |
| Model 3 | 1.00 (ref.) | 1.08 (0.67-1.74) | 1.42 (0.96-2.10) | **1.76 (1.14-2.74)**** | **0.008** |
| ASCVD | | | | | |
| n/N | 31/1708 | 27/1667 | 60/2065 | 42/1324 | |
| Model 1 | 1.00 (ref.) | 1.16 (0.69-1.94) | 1.47 (0.96-2.27) | **2.04 (1.28-3.26)**** | **0.002** |
| Model 2 | 1.00 (ref.) | 1.18 (0.70-2.00) | 1.45 (0.94-2.24) | **1.99 (1.25-3.18)**** | **0.003** |
| Model 3 | 1.00 (ref.) | 1.19 (0.70-2.02) | 1.43 (0.92-2.24) | **1.97 (1.20-3.21)**** | **0.007** |
| Stroke | | | | | |
| n/N | 34/1708 | 31/1667 | 66/2065 | 40/1324 | |
| Model 1 | 1.00 (ref.) | 1.20 (0.73-1.96) | 1.48 (0.98-2.24) | **1.77 (1.12-2.81)*** | **0.008** |
| Model 2 | 1.00 (ref.) | 1.22 (0.74-2.00) | 1.45 (0.96-2.20) | **1.72 (1.09-2.73)*** | **0.014** |
| Model 3 | 1.00 (ref.) | 1.25 (0.75-2.06) | 1.40 (0.92-2.14) | **1.65 (1.02-2.66)*** | **0.039** |
| Ischaemic stroke | | | | | |
| n/N | 25/1708 | 25/1667 | 50/2065 | 33/1324 | |
| Model 1 | 1.00 (ref.) | 1.32 (0.75-2.30) | 1.52 (0.94-2.46) | **1.99 (1.18-3.35)**** | **0.009** |
| Model 2 | 1.00 (ref.) | 1.40 (0.79-2.46) | 1.50 (0.93-2.44) | **1.98 (1.17-3.34)*** | **0.012** |

| | | | | | |
|---|---|---|---|---|---|
| Model 3 | 1.00 (ref.) | 1.45 (0.81-2.57) | 1.45 (0.89-2.38) | **1.86 (1.07-3.22)*** | **0.033** |
| CHD | | | | | |
| n/N | 9/1708 | 3/1667 | 13/2065 | 12/1324 | |
| Model 1 | 1.00 (ref.) | 0.49 (0.13-1.69) | 1.13 (0.48-2.65) | 2.09 (0.88-4.97) | 0.069 |
| Model 2 | 1.00 (ref.) | 0.44 (0.12-1.67) | 1.05 (0.45-2.48) | 1.87 (0.78-4.49) | 0.103 |
| Model 3 | 1.00 (ref.) | 0.39 (0.10-1.49) | 1.06 (0.44-2.55) | 1.96 (0.78-4.97) | 0.142 |

Abbreviations: HR, hazard ratios; CI, confidence interval.
CVD, cardiovascular disease; ASCVD, atherosclerotic cardiovascular disease; CHD, coronary heart disease.
Model 1: adjusted for age(continuous variable), sex
Model 2: model 1 plus present smoking status, excessive drinking status, physical activity, cereal intake, vegetables and fruits intake, bean products intake, Animal entrails intake, and excessive meat intake.
Model 3: model 2 plus BMI(continuous variable), Lipid-lowering drugs use, LDL-C(continuous variable), Diabetic nephropathy, diabetes, hypertension.
*$P$<0.05;**$P$<0.01

Table S7 The incident risk of CVD Outcomes associated with baseline concentration of Remnant Cholesterol (continuous variable).

| Outcomes | HR (95% CI) | | | |
|---|---|---|---|---|
| | Events | Model 1 | Model 2 | Model 3 |
| CVD | 199/6764 | **1.28 (1.07-1.53)**** | **1.26 (1.06-1.51)*** | **1.24 (1.02-1.51)*** |
| ASCVD | 160/6764 | **1.31 (1.08-1.59)**** | **1.30 (1.07-1.58)**** | **1.27 (1.03-1.58)*** |
| Stroke | 171/6764 | **1.24 (1.02-1.50)*** | **1.23 (1.01-1.49)*** | 1.18 (0.96-1.46) |
| Ischemic stroke | 133/6764 | **1.27 (1.02-1.57)*** | **1.26 (1.01-1.56)*** | 1.21 (0.95-1.53) |
| CHD | 37/6764 | **1.51 (1.03-2.22)*** | **1.49 (1.00-2.22)*** | 1.51 (0.97-2.35) |

Abbreviations: HR, hazard ratios; CI, confidence interval.
CVD, cardiovascular disease; ASCVD, atherosclerotic cardiovascular disease; CHD, coronary heart disease.
Model 1: adjusted for age(continuous variable), sex
Model 2: model 1 plus present smoking status, excessive drinking status, physical activity, cereal intake, vegetables and fruits intake, bean products intake, Animal entrails intake, and excessive meat intake.
Model 3: model 2 plus BMI(continuous variable), Lipid-lowering drugs use, LDL-C(continuous variable), Diabetic nephropathy, diabetes, hypertension.
*$P$<0.05;**$P$<0.01
